# Complex malaria epidemiology in an international border area between Brazil and French Guiana: challenges for elimination

**DOI:** 10.1186/s41182-019-0150-0

**Published:** 2019-04-11

**Authors:** Vivian da Cruz Franco, Paulo Cesar Peiter, José Joaquim Carvajal-Cortés, Rafael dos Santos Pereira, Margarete do Socorro Mendonça Gomes, Martha Cecilia Suárez-Mutis

**Affiliations:** 1 Laboratory of Parasitic Diseases, Institute Oswaldo Cruz/Fiocruz, Av Brasil 4365. Pavilhão Arthur Neiva, Rio de Janeiro, RJ 21040-900 Brazil; 2 Biology of Infectious and Parasitic Agent, UFPA, Superintendence of Health Surveillance, Goverment of Amapá State, Rua Tancredo Neves, 1118. São Lázaro, Macapá, AP 68908530 Brazil

**Keywords:** Malaria, Elimination, Oiapoque, Illegal gold miners, Amazon

## Abstract

**Background:**

The epidemiological surveillance of malaria is a necessary intervention for eliminating the disease from the planet. The international border zones of the Amazon continue to be highly vulnerable to malaria since population mobility impedes elimination. Although in the past few years, cases of malaria have had an essential reduction in Brazil, this trend was not confirmed in municipalities along the border. This study aimed to establish the epidemiology of the disease during the last 13 years in Oiapoque, a Brazilian municipality at the international border with French Guiana, an overseas department, to develop strategies for the control/elimination of malaria in these areas.

**Results:**

Data collected from 2003 to 2015 from the Malaria Epidemiological Surveillance System was used. It was found that, despite the important reduction in cases (68.1%), the annual parasite index remained a high epidemiological risk. The disease is seasonal in that the period of highest transmission occurs between September and December. Between 2003 and 2015, eight outbreaks were identified, with one of these lasting 15 months between August 2006 and October 2007. There were changes in the epidemiological profile, with imported cases representing 67.7% of cases from 2003 to 2007 and representing 32.9% of cases from 2008 to 2015 (*p* < 0.01). The greatest number of cases was among Brazilians coming from the artisanal gold mines of French Guiana. There were also changes in the profile of autochthonous malaria with an increase in urban cases from 14.3% in 2003 to 32.3% in 2015 (*p* < 0 .01). The burden of malaria in indigenous areas was also very high (67.3% in rural areas) in 2015. There were changes in the parasite species profile with a significant decrease of cases of *Plasmodium falciparum* (*p* = 0.01). Children under 15 years old, representing 9.7% of cases at the onset of the study, accounted for 34.2% of case notifications (*p* < 0.01) in 2015. Also, 74% of cases in 2003 and 55.9% in 2015 (*p* < 0.01) were among men.

**Conclusions:**

The fragility of local health services in cross-border areas continues to be an obstacle for malaria elimination.

**Electronic supplementary material:**

The online version of this article (10.1186/s41182-019-0150-0) contains supplementary material, which is available to authorized users.

## Background

The international border of the Amazon is the most vulnerable portion of the Brazilian border and has been affected by a serious public health problem: malaria that is endemic in the entire region [1, 2]. In this area, both the surveillance and elimination of this disease are important challenges, as cross-border mobility is part of the daily life of residents, hampering control efforts [3]. Brazil was one of the countries that managed to reduce the burden of malaria in 2015 compared with 2000, reaching one of the millennium goals with a 76.7% reduction of cases [4]. The Ministry of Health observed a reduction in the number of cases of the disease in the majority of Amazonian municipalities with the transmission, but the same did not occur in areas along the international border. At a time when the planet is dedicated to eliminating malaria, the failure to control the borders of countries is a concern for health authorities, as areas, where the disease has been eliminated, have the risk of reintroduction of disease through imported cases. Since 2015, Brazil has accepted the challenge of eliminating *Plasmodium falciparum,* in addition to the challenges of the complex epidemiological context of the country [5].

In the border area between Brazil and French Guiana, multiple factors have contributed to the occurrence of malaria, but in-depth research is needed to understand its main determinants to identify the best possible measures of disease control. This study analyzed the epidemiology of malaria in this border area to propose strategies for the control and/or elimination of the disease in this area.

## Results

**Fig. 1:**
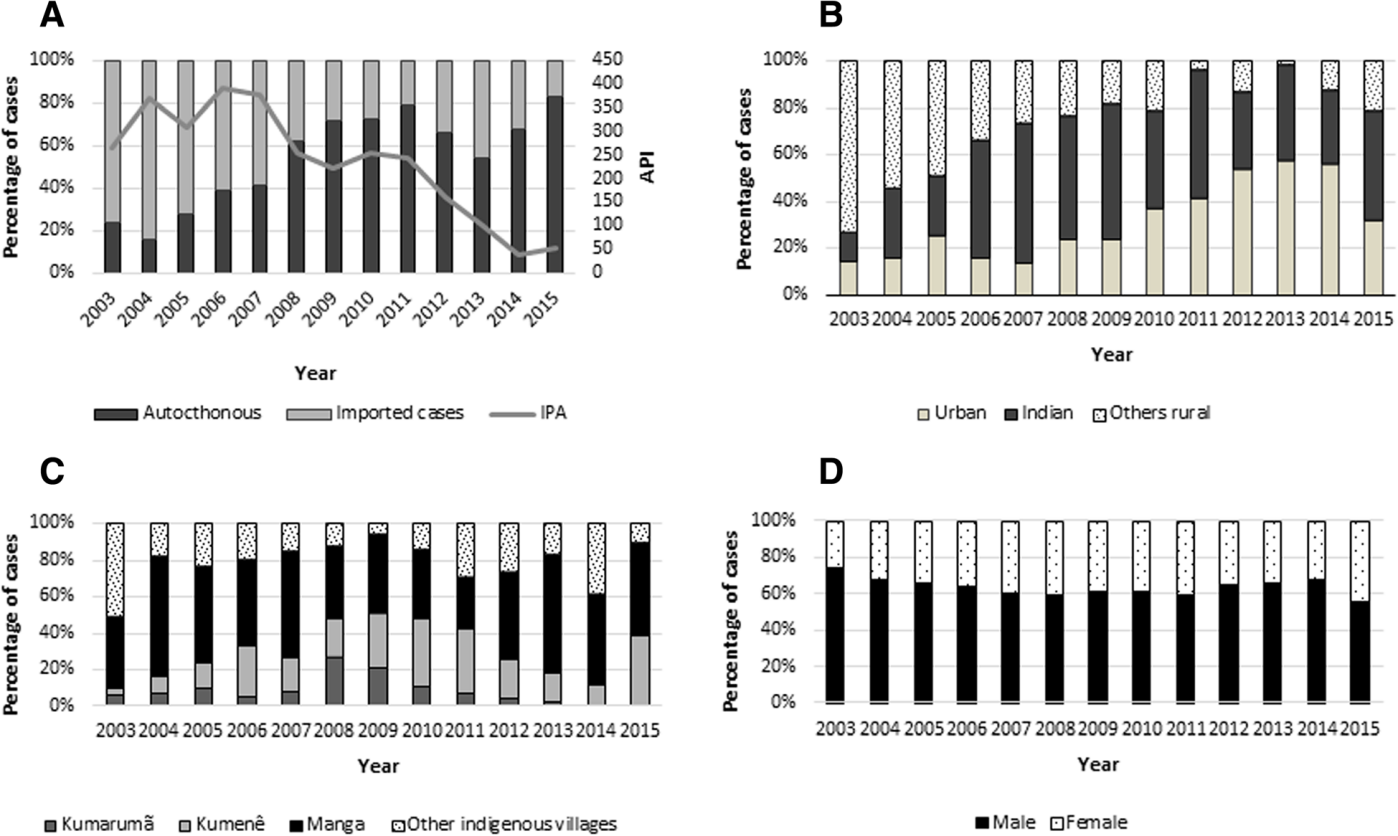
**a**. Distribution of imported and autochthonous cases between 2003 and 2015 and API during the study period. **b**. Distribution of urban, indigenous and other rural cases during the study period. **c**. Distribution of indigenous cases by base poles and other villages. **d**. Proportion of malaria cases between men and women between 2003 and 2015

From January 2003 to December 2015, 55,194 cases of malaria were identified in the municipality of Oiapoque; the annual average between 2003 and 2015 of reported cases was 4.25 ± 1.84 (95% CI 3.13–5.36), with a median of 4698 reported cases. In 2003, 3877 cases were reported, and in 2015, there were 1236, a reduction of 68.1%. The highest number of reported cases was in 2004, 2006, and 2007 (5577, 6373, and 6593, respectively). The average Annual Parasite Index (API) was 234.9 cases per 1000 inhabitants (standard deviation 117.6) between 2003 and 2015 and experienced a substantial reduction as of 2012: in 2003, the API was 266.9 per 1000 inhabitants and, in 2015, the API was 52.3 cases per 1000 inhabitants. Despite the reduction in the API, it remained a high epidemiological risk throughout the analyzed period (Fig. 1a).

**Fig. 2:**
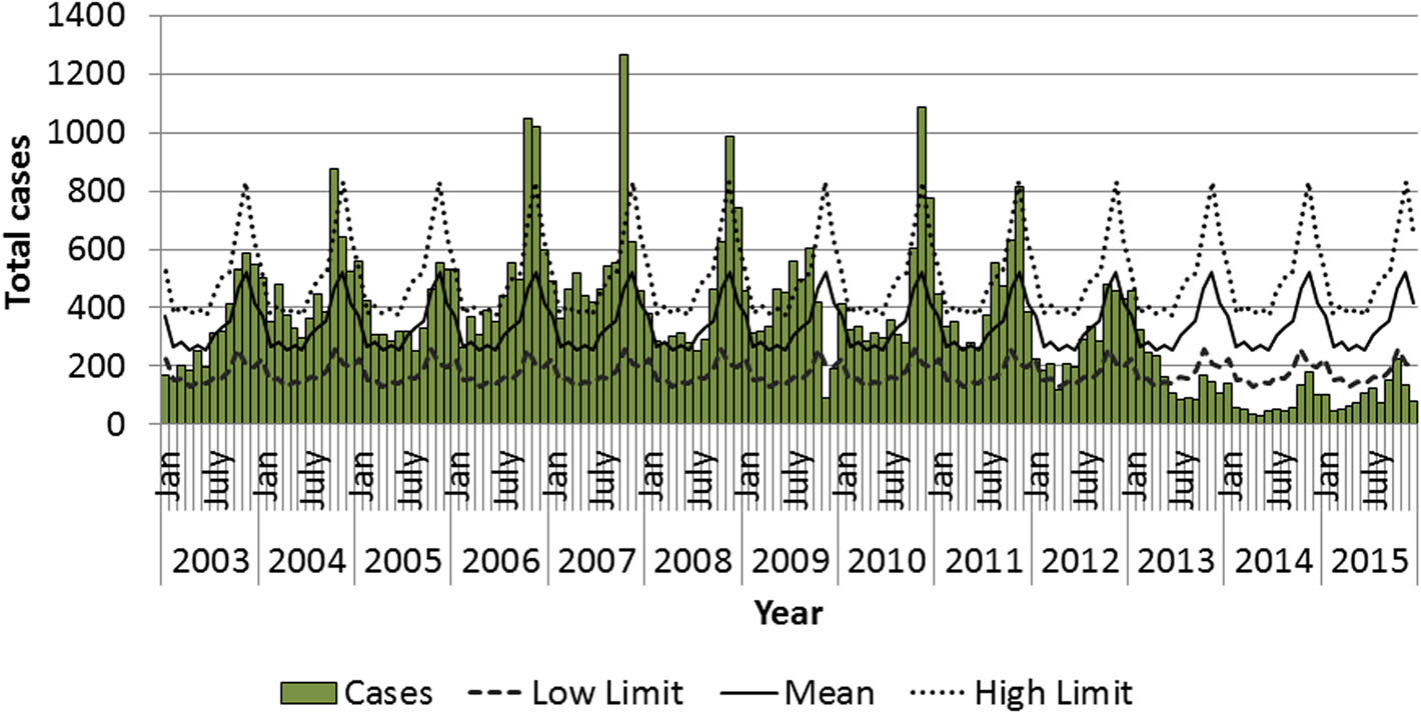
Endemic curve of malaria cases in the municipality of Oiapoque between 2003 and 2015. Source: Sivep-Malaria 2016

Figure 2 presents the endemic curve of cases of malaria in the municipality between 2003 and 2015. Cases of malaria were reported every month, but, as of September, the number of cases would increase, reaching their peak in October and November. The beginning of transmission season coincides with the dry period that is from September until December. From 2004 to 2011, eight outbreaks were observed: an epidemic that began in August 2006 and continued until October 2007 should be noted. This year was considered an epidemic year since the number of cases surpassed the upper limit in almost every month of 2007.

## Imported cases

Of reported cases, 28,326 (51.3%) were autochthonous and 26,858 (48.7%) were imported from other municipalities or countries. Two periods can be observed: the first from 2003 to 2007 and the second from 2008 to 2015 (Fig. 1a and b). In the first period, 67.7% of case notifications were imported cases from another country, 1.4% was imported from another municipality, and 30.9% were autochthonous cases. As of 2008, there was a change in the pattern of case notifications with a decrease in the number of imported cases and an increase in autochthonous cases. From 2008 to 2015, 66.7% of cases were autochthonous, 32.9% were imported from other countries, and 0.4% imported from another municipality. The changes in percentages of cases imported from other countries and other municipalities in the two periods were statistically significant (*p* < 0.01 and *p* < 0.05, respectively).

French Guiana was the country which exported the most cases to the municipality of Oiapoque between 2003 and 2015, with 98.1% of internationally imported cases coming from that country. The majority of these cases were among artisanal gold miners working in illegal mines in French Guiana and/or Suriname, where they were infected. Of cases received from French Guiana, 40.1% (10,710/26,733) were of *Plasmodium falciparum*.

### Autochthonous cases

Of autochthonous cases, 8803 (31.1%) originated in urban areas and 19,523 (68.9%) in rural areas (Fig. 1b). In 2003, 14.3% (134/938) of cases were urban and 85.7% (804/938) were rural. In 2012, urban areas accounted for 53.7% (1269/2365) of cases and rural areas accounted for 46.7% (1096/2365). As of 2013, there was a decrease in case notifications, and in 2015, 32.3% (333/1029) of cases were registered in urban areas. This difference was statistically significant (*p* < 0.01).

### Urban malaria

The average number of urban cases between 2003 and 2007 was 277 (95% CI 117–437, median 335). Between 2008 and 2015, the average number of urban cases was 927 (95% CI 478.4–1376, median 792). This difference was statistically significant (*p* = 0.03). At the start of the study in 2003, only 14.3 of the autochthonous cases were of urban malaria. As of 2008, this percentage changed, reaching 57.7% of cases in 2013. In 2015, there was a change in the pattern, and urban cases were 32.7% of notifications (333/1030). An analysis of the series of cases during the period indicated that the number of notifications increased over the years, except for 2015, when it decreased (Fig. 1b).

**Fig. 3:**
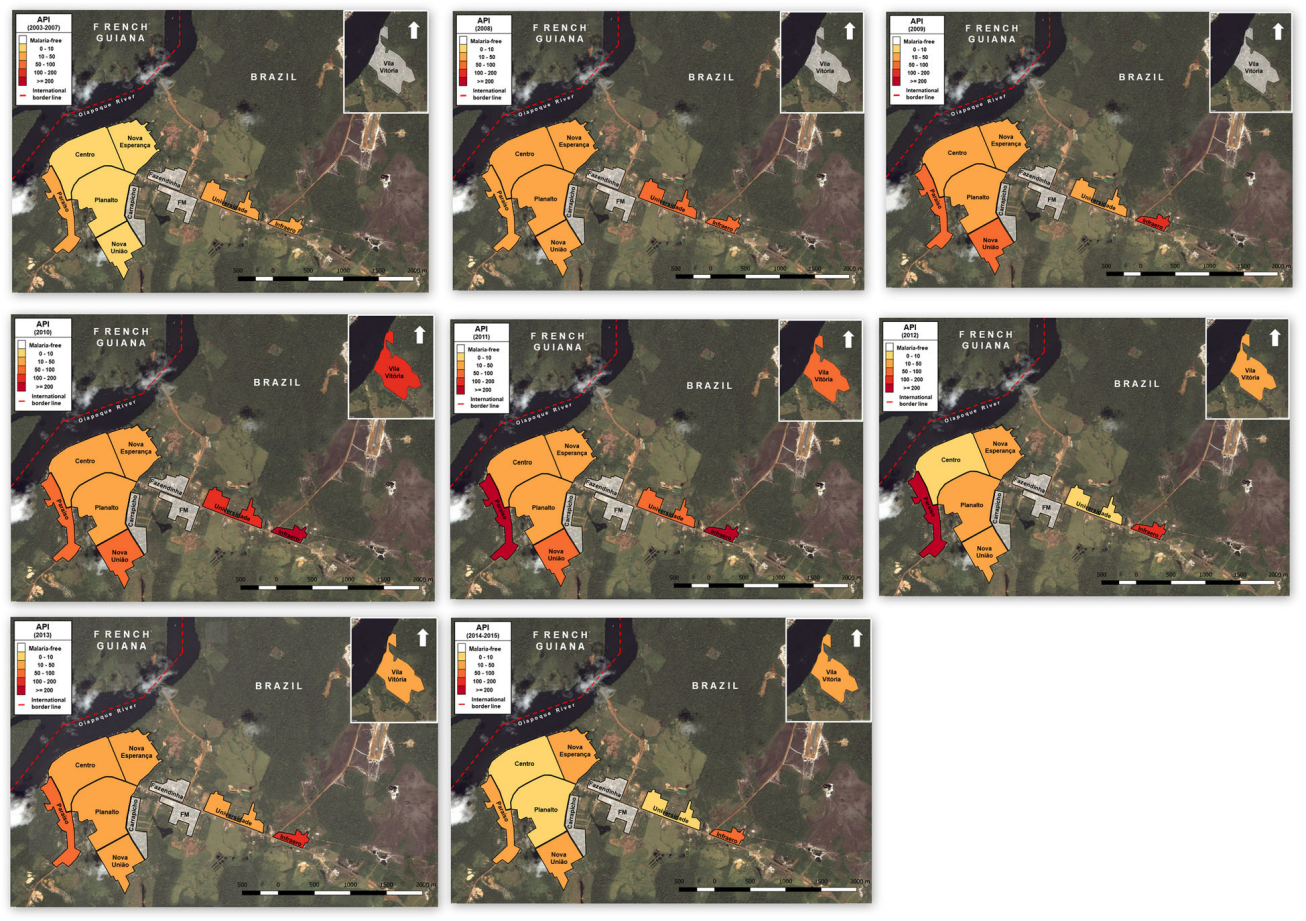
Spatial distribution of the incidence of malaria in the urban areas of the municipality of Oiapoque between 2003 and 2015. Source: Sivep-Malaria 2016

Figure 3 presents the spatial distribution of the API in urban areas of Oiapoque from 2003 to 2015. Although there was variation in the distribution of cases in every district throughout the study period, the districts of Paraiso and Infraero always presented an important number of cases. The districts of Fazendinha, Carrapicho, and FM did not have any registered case notifications. Their absences may have occurred because they are not official territories and are not entered in SIVEP-Malaria.

From 2003 to 2007, the districts with the highest API were Infraero, Paraíso, and Universidade with 29.1, 21.2, and 20.1 cases per 1000 inhabitants, respectively. From 2008 to 2013, the districts with the highest number of cases were Infraero and Paraíso with 165.6 and 142.6, respectively. The case notifications from Vila Vitória began in 2010, with an API of 125 per 1000 inhabitants. From 2014 to 2015, Infraero and Paraíso presented an average API of 58.2 and 51.1, respectively (Fig. 3).

### Malaria in rural areas

Malaria in rural areas had two components: indigenous areas and non-indigenous rural areas (Fig. 1b). During the period from 2003 to 2015, 13,142 cases of malaria occurred among the indigenous people, representing 41.9% of the total number of autochthonous cases in the municipality, and 67.3% of rural cases. Unlike what happened with imported malaria, there appears to have been three moments of malaria in indigenous areas: between 2003 and 2005, the average number of cases was 240.7 ± 112; between 2006 and 2011, it was 1755 ± 386, and, between 2012 and 2015, it was 470.8 ± 240. The average API in the indigenous areas during the study period was 151.1/1000 inhabitants. In 2003, this API was 33.1/1000 inhabitants, reaching 72.7/1000 inhabitants in 2015. In 2015, malaria in the indigenous region was responsible for 68.8% of the disease in the rural area. The most considerable part of notified cases was registered within the Manga coverage area, which serves the highest number of indigenous villages (Table 1, Fig. 1c and Additional file 1). The API in 2015 in the Manga coverage area was 145.2 cases per 1000 inhabitants; in Kumenê, it was 107.8 cases per 1000 inhabitants; and in Kumarumã, it was 1.5 cases per 1000 inhabitants. The remaining villages had an API of 254.7 per 1000 inhabitants.

**Table 1:** Cases of malaria reported in the municipality of Oiapoque, Amapá, Brazil, according to geographical area of origin, 2003–2015

Year	Urban		Rural												Total case
			Polo Kumarumã		Polo Kumenê		Polo Manga		Other villages		Other rural areas		Rural total		
	*n*	%*	*n*	%**	*n*	%**	*n*	%**	*n*	%**	*n*	%**	*n*	%*	
2003	134	14.3	7	0.9	5	0.1	46	5.7	60	7.5	686	85.3	804	85.7	938
2004	142	16.0	20	2.7	24	3.2	174	23.4	48	6.5	477	64.2	743	84.0	885
2005	335	25.5	35	3.6	47	4.8	176	17.9	80	8.2	643	65.5	981	74.5	1316
2006	395	15.9	64	3.1	353	16.9	572	27.3	255	12.2	850	40.6	2094	84.1	2489
2007	380	13.8	134	5.7	309	13.1	934	39.5	254	10.7	735	31.1	2366	86.2	2746
2008	769	23.8	449	18.2	368	15.0	675	27.4	208	8.5	761	30.9	2461	76.2	3230
2009	815	24.1	418	16.3	586	22.8	839	32.6	113	4.4	616	24.0	2572	75.9	3387
2010	1451	37.4	184	7.6	587	24.2	611	25.2	226	9.3	818	33.7	2426	62.6	3877
2011	1822	41.6	178	7.0	833	32.5	684	26.7	694	27.1	171	6.7	2560	58.4	4382
2012	1269	53.7	33	3.0	169	15.4	375	34.2	204	18.6	315	28.7	1096	46.3	2365
2013	606	57.7	13	2.9	67	15.1	272	61.3	72	16.2	20	4.5	444	42.3	1050
2014	352	55.7	2	0.7	22	7.9	98	35.0	77	27.5	81	28.9	280	44.3	632
2015	333	32.4	2	0.3	186	26.7	238	34.2	53	7.6	217	31.2	696	67.6	1029
Total	8803	31.1	1539	7.9	3556	18.2	5694	29.2	2344	12.0	6172	31.6	19,523	68.9	28,326

*Percentage of total municipality

**Percentage of total rural area

The remaining cases of the rural area (6390 cases, 32.7% of the total) occurred in different locations. It should be noted that a location known as “Ramal km 8,” of only 44 inhabitants, was the site of 590 (86%) of the 686 cases in the rural non-indigenous area in 2003. This same location significantly contributed to the malaria burden over several years. In 2015, in this area, malaria was considered extinct and only two cases of malaria were reported. Other rural locations also presented moments with important numbers of cases: of which, Ilha Bela and Vila Brasil, areas used for relaxation and shopping among gold miners along the Oiapoque River, stand out.

**Fig. 4:**
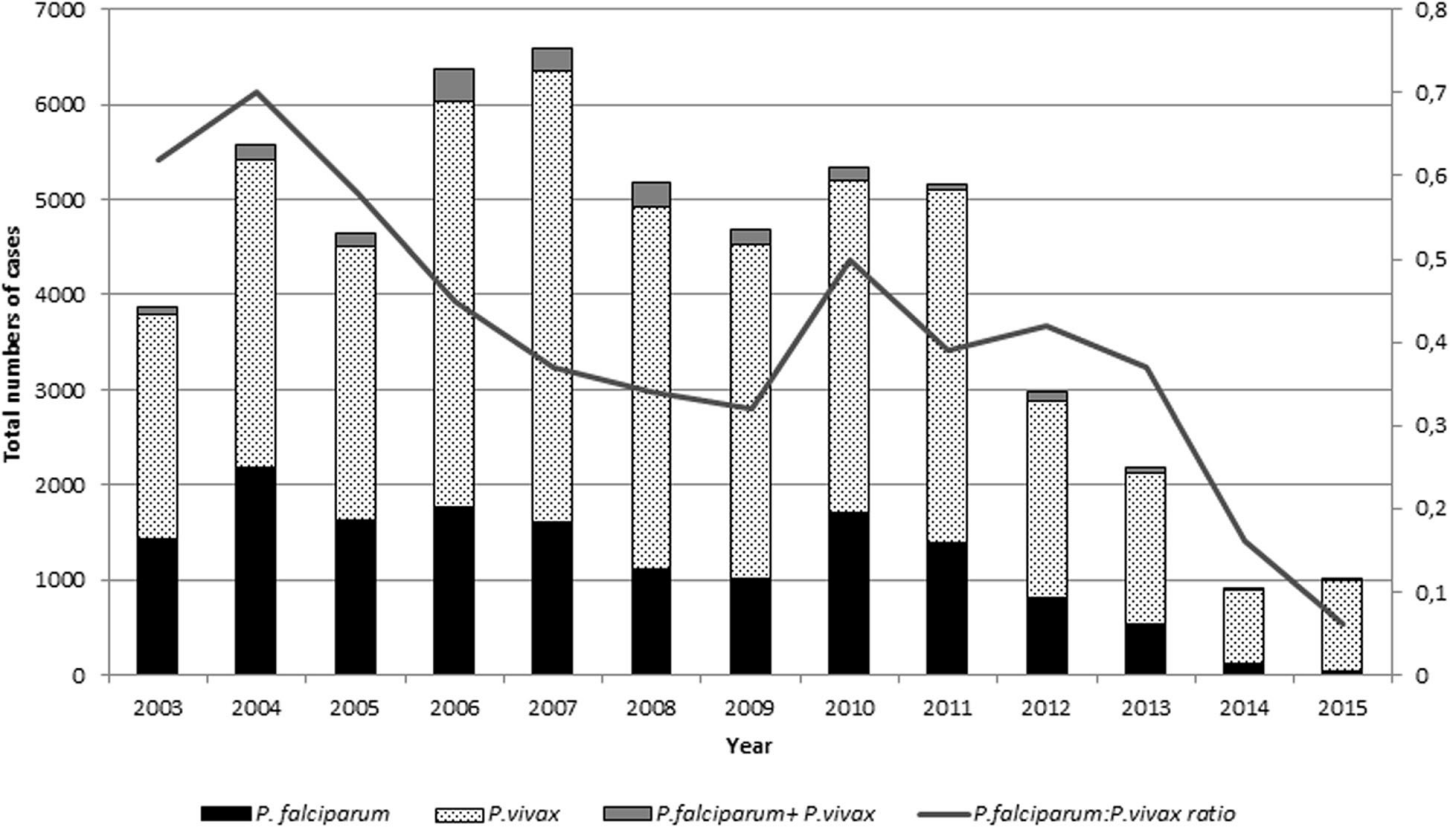
Proportion of cases by parasite species and rate of *Falciparum* + *vivax* between 2003 and 2015. Source: Sivep-Malaria 2016

The predominant parasite species during the study period was *P. vivax*, 67.8%, followed by *P. falciparum*, 27.8%, mixed infections with *falciparum + vivax* (3.2%), and *P. malariae*, 0.2%. When evaluating the two periods, the average number of cases of *P. falciparum* from 2003 to 2007 was of 1909 cases/year (95% CI 1508–2310, median = 1836, total 9544), and from 2008 to 2015, the average was 945 cases/year (95% CI 408.9–1481, median = 1041, total 7561). These differences were statistically significant (*p* = 0.01). The average number of *P. vivax* cases between 2003 and 2007 was of 3684, and between 2008 and 2015, it was 2595; but, this difference was not significant (*p* = 0.28) (Fig. 4). There was a difference in the *P. falciparum* to *P. vivax* ratio.

**Fig. 5:**
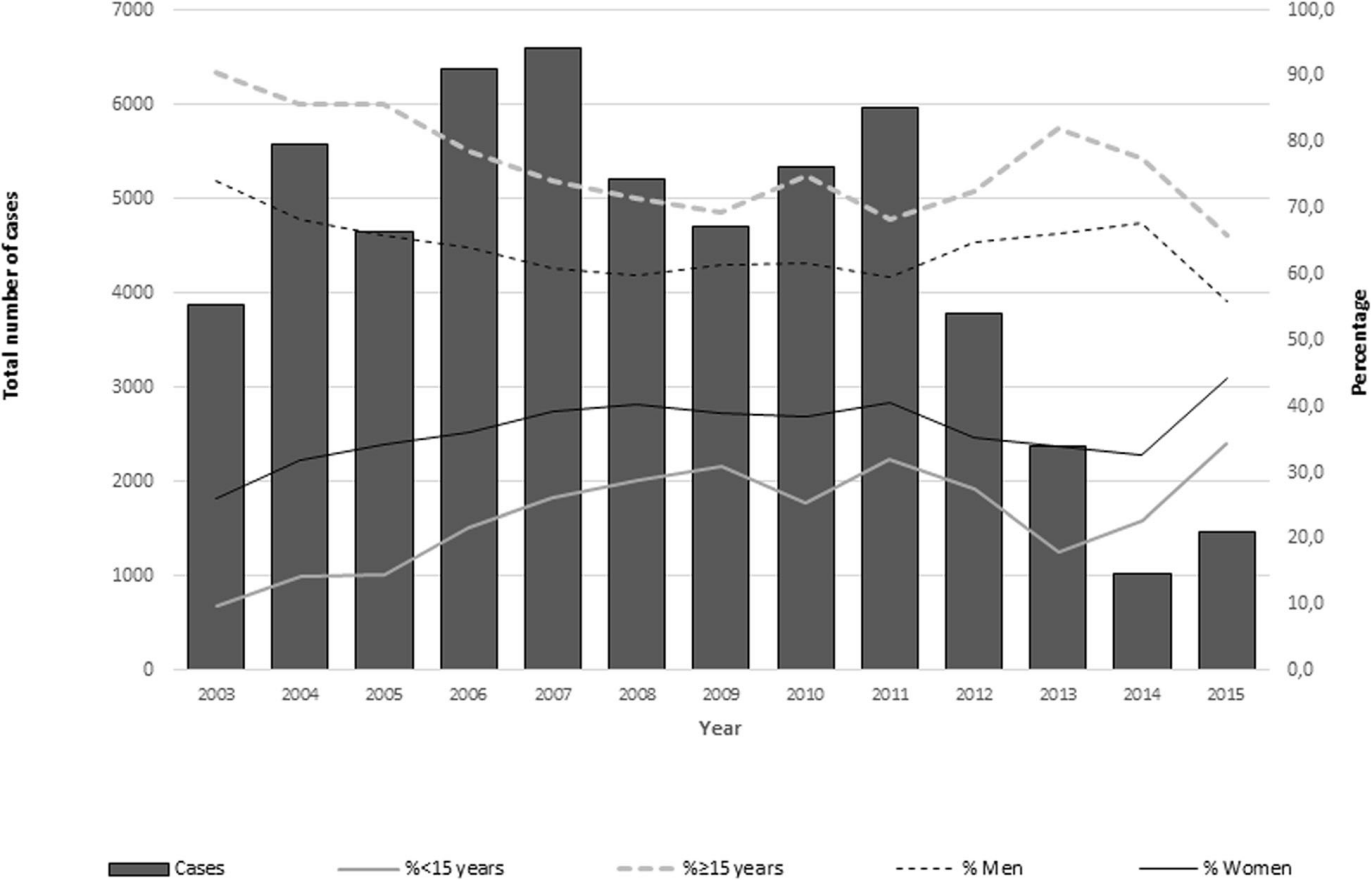
Cases of malaria and rate of proportion of men and individuals under 15 years between 2003 and 2015

During the study period, the most affected age group was from 15 to 49 years, which accounted for 73.3% of the total positive cases. A change was observed in the structure of the age groups most affected by the disease: at the start of the period, in 2003, 85.8% of the reported cases were among adults between 15 to 49 years; in 2015, it was 60.8% (*p* < 0.01). In contrast, children under 15 years were 9.7% of cases in 2003, and a higher disease burden in 2015 when they were 34.2% of cases (*p* < 0.01) (Fig. 5).

Regarding sex, in the period from 2003 to 2015, 64.5% (35,702/55,319) of cases of malaria in the municipality of Oiapoque were among men, and 35.5% (20,414/55,319) were among women. If 2003 and 2015 are compared, although the greatest number of registered cases was still among men, the percentage decreased in comparison with cases among women. In 2003, 74% of cases were among men (2868/3877), and in 2015, they represented 55.9% of cases (679/1236). This difference was statistically significant (*p* < 0.01) (Fig. 5).

## Discussion

During the last few years, the planet has been experiencing a renewed interest in eliminating malaria. There are still many challenges, which must be overcome, and knowledge of different epidemiological scenarios as well as characterization of transmission profiles and various ecosystems, which may harbor malaria, is fundamental in this struggle [6]. This study aimed to establish different transmission scenarios of malaria in a municipality along the international border of Brazil and French Guiana. The municipality of Oiapoque is considered a high epidemiological risk area for malaria. It can be defined as an unstable region with endemic malaria and prone to outbreaks. Transmission varies both from month to month and year to year, depending on many factors, such as illegal miners who cross the border with French Guiana and malaria among the indigenous population and in urban areas of the municipality. The burden of malaria among these different scenarios has changed over the study period.

During the last few years, many studies have shown malaria trends in both the Americas and Brazil [7–9]. The study by Lima et al. examined differences between states, demonstrating the heterogeneity of epidemiological scenarios. In 2015, Brazil achieved a total reduction of 76.7%, the lowest number of cases in the last 35 years. This achievement led the Ministry of Health to launch the “Plan to Eliminate Malaria with *P. falciparum*” in 2016 [5, 10]. However, this decrease was not seen equally in every state. In Amapá, there was also a reduction of malaria cases, but this reduction was limited in comparison with other Amazonian states [9]. It is suspected that the decrease was not more significant in the state because of the contribution of cases of malaria from the municipality of Oiapoque, bordering French Guiana. The presence of illegal mines, an unstable health system, and a constant flow of people crossing the border into this municipality partly explain its role in contributing to the burden of malaria in the state [9]. Despite a meaningful reduction in the number of cases between 2000 (2754 cases) and 2015 (1236 cases), Oiapoque did not reach the millennium goal of reducing malaria by 75%, instead only reaching a 55.1% reduction [11].

**Fig. 6:**
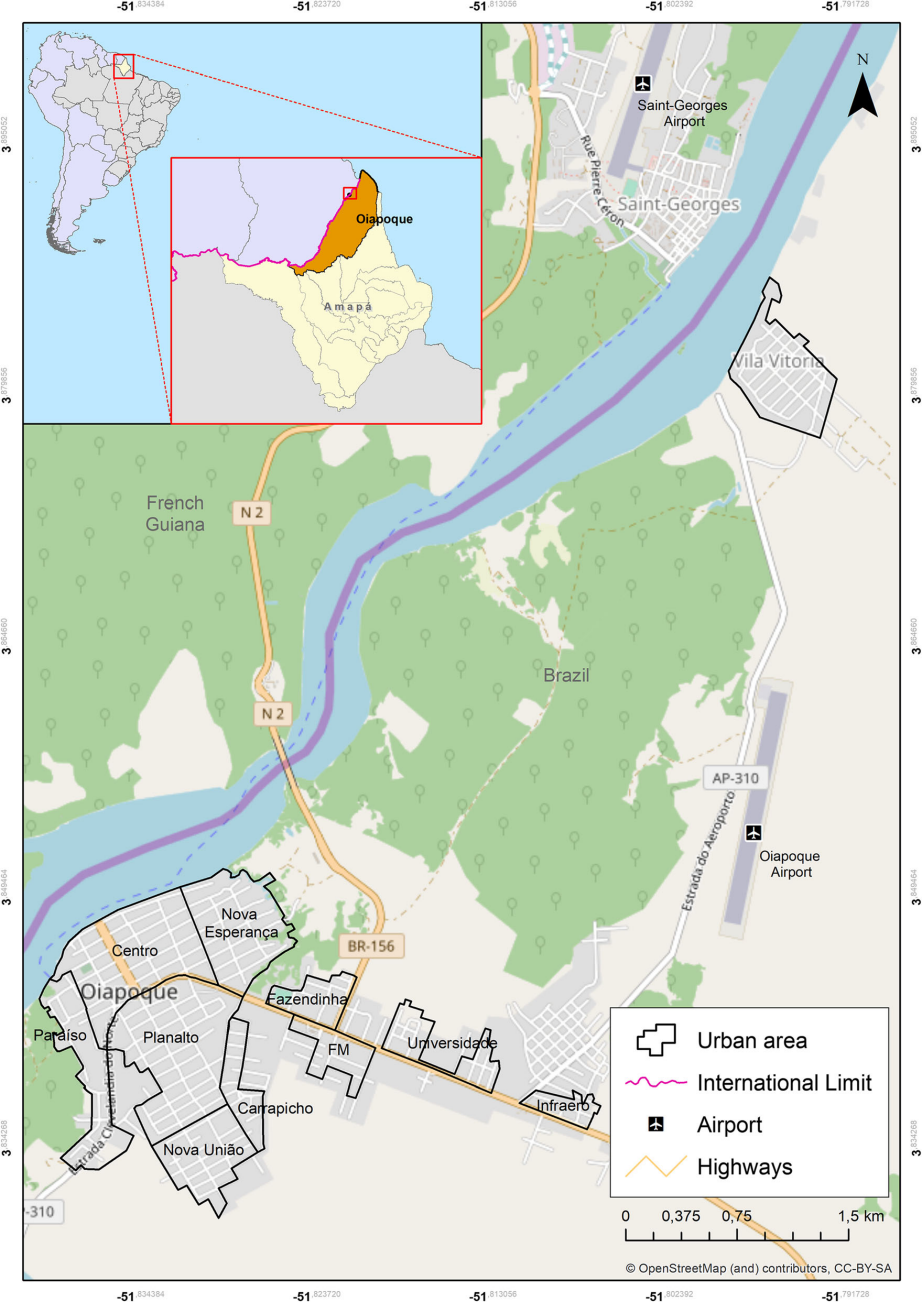
Geographic location the municipality of Oiapoque-Amapá State Brazil

The dynamics of the disease during the 13 years studied demonstrate changes in malaria in Oiapoque. First, there were changes to the percentage of imported cases and autochthonous cases of the disease: the imported cases in 2003 were 75.8% of the total, and in 2015, they were only 16.7%. This change was statistically significant. Oiapoque was the municipality with the highest number of internationally imported cases of malaria. A study from 2003 to 2010 of the municipalities along the Brazilian border recognized that these municipalities reported an average of 13.8% of imported cases, with Oiapoque, AP (55.5%) receiving the highest percentage of internationally imported cases of malaria [12]. From January to August 2017, French Guiana accounted for 12% of imported cases. The high number of imported cases received in Oiapoque, especially from 2003 to 2007, should be noted. These cases of malaria result from cases among Brazilian gold miners, without a doubt, one of the most critical determinants of disease in this municipality. The gold mines, most of them illegal, are in French Guiana and Suriname, but some are in Guyana and Amapá State (Fig. 6). It is estimated that 20,000 people, mostly Brazilians, work in the mines in Suriname, and 7000 in French Guiana [13]. Most cases in Oiapoque were among people, who worked in French Guiana or Suriname, were infected working in illegal mines and returned ill to Brazil for treatment. Late diagnosis and treatment are the reality among this population. In the French territory, malaria is precisely determined by miners with 80% of cases occurring along the border with Suriname and 20% along the border with the Oiapoque River [13]. Juminer et al. (1981) [14] demonstrated that in the 1980s, the incidence of disease along the Oiapoque River, on the French side of the border, was 485 cases per 1000 inhabitants, with 30% of cases attributed to *P.* *vivax*. In 2013, cases of malaria in Saint-Georges-de l’Oyapock and Camopi were 55.2/1000 inhabitants and were considered high epidemiological risk and associated with illegal mines [15, 16]. Since 2002, a series of operations have been conducted by the French armed forces to both control and reduce illegal mines in forested areas of French Guiana, leading miners to abandon these areas. During these operations, the transmission of malaria decreased in French Guiana [17, 18]. However, the municipality of Oiapoque has served as a major receiver of this population. Since it is an illegal activity, minimal information exists about malaria in the mines. In the malaria information system in Brazil (SIVEP-Malaria), case notifications regarding mining areas only existed as disaggregated data as of 2014: the area known as Crikou contributed 41 and 25 cases in 2014 and 2015, respectively, being that this is an exceptionally small number in comparison with existing data. Given the illegality of mining, many people do not give correct information regarding the probable infection location out of fear.

Douine et al. (2016) [19] conducted a study with people involved with mines along the border between French Guiana and Suriname. They found that 22.3% (95% CI [18.3–26.3], *n* = 94/421) of miners were infected and, of those, 84% were asymptomatic. This population is extremely vulnerable because, besides malaria, many other diseases weaken their health [20]. This information is extremely valuable because it demonstrates that this difficult to access population, in addition to the humanitarian concern of their prolonged illness, can act as a parasite reservoir. Their lack of adherence to antimalarial treatment can produce resistant strains, hindering efforts to eliminate malaria.

Another aspect worth highlighting is the change in the epidemiological profile of malaria with an increase of urban cases and a decrease in rural areas. At the start of the study period, the percentage of rural cases was 85.7%; by 2014, the rural area presented 44.3% of cases in the municipality; and, in 2015, it accounted for 67.6% of cases, making these changes statistically significant. The factors that determined the change in disease pattern should be better evaluated. It is probable that the expulsion of Brazilians from the mines in French Guiana could have been a determining factor in the increase in urban malaria in Oiapoque since the periphery of this city was the reception area for this population [21]. While, in Brazil, malaria in urban areas decreased by 23% between 2003 and 2011 [22], in Oiapoque, there was an increase in this percentage. The uncontrolled occupation of peri-urban spaces is, without a doubt, an essential determinant of malaria in the Amazon region. The main areas where malaria is present in cities are those with precarious living conditions, which do not represent truly urban spaces [23, 24]. It is necessary to note that although malaria has been present in nearly every district of the municipality, the highest concentration of cases consistently occurred in the districts of Paraíso and Infraero. The district of Paraíso, located near the city center, is surrounded by the Igarapé do Palha River (a little river) and suffers directly with floods and tides of the river. These water collections create the conditions for both the proliferation of the vector and emergence of larval habitats. The district of Infraero, near the woods and belonging to the Brazilian Company of Airport Infrastructure (Infraero), began to be inhabited a little over 10 years ago and has undergone recurring occupations. Vila Vitória is located along the Oiapoque River, in front of the city of Saint-Georges-de-l’Oyapock (a municipality in French Guiana). This district was developed about 11 years ago and, therefore, does not have notifications of malaria before 2009. It has a curious history narrated by its residents: 25 people in search of employment opportunities in French Guiana were deported from the French territory by the police and were prohibited from returning. These men and women united to occupy the private property on which they settled, creating this settlement without any planning. The streets contain slopes, many of which are unpaved, without sanitation, and with precarious trash collection services. This disorganized occupation, which kept the population very near the forest, favors the proliferation of vector-borne diseases, such as malaria and dengue, among others [25].

Another important observation was the increase in malaria among the indigenous population. The indigenous villages in the municipality present some peculiarities: Manga Village, for example, is accessible through route 156 allowing the inhabitants to reach the municipal center with greater ease, but it takes 5 hours of travel by boat along various rivers to reach the Kumarumã Village. The indigenous residents of Kumenê Village are even more isolated: to arrive at this community, 20 h of travel along the Oiapoque, Uaçä, and Urukauá Rivers are needed, and travel may take even longer depending on the tides. Distances are a challenge for health care providers. Despite the problem of malaria in these communities, unfortunately, during the past few years in Brazil, policies for the indigenous population have suffered a crisis. With the administrative decentralization of malaria control, programs to the municipalities and transfer of indigenous health (originally under the National Foundation of Health—FUNASA) in 2005 to the Secretariat of Indigenous Health (SESAI), no one assumes ongoing responsibility for malaria in these areas.

The high number of cases of *P. falciparum* (Pf) in Oiapoque at the beginning of the study and the critical reduction of cases throughout the period should be noted. In 2003, this species caused 38.9% of the total cases of malaria. In 2005, an epidemic year in Brazil, infections with *P. falciparum* accounted for 25% of malaria cases. In this same year, cases of Pf in Oiapoque were 30% of the total. Similarly, there was a substantial reduction in the number of cases of *P. falciparum* since the beginning of the study. A large portion of malaria with *P. falciparum* can probably be attributed to miners who worked in French Guiana or especially in Suriname, where *P. falciparum* is the dominant species [26, 27]. With the introduction of artemisinin-based combination therapy in late 2006, reported cases in Brazil fell to less than 12% [24]; in Oiapoque in 2015, *P. falciparum* accounted for only 4.5% of cases, including mixed malaria cases (Pv + Pf). These treatment schedules are not only more effective but also allow for greater patient adherence to drugs because they need fewer doses and less time to complete treatment. Another factor that may have influenced the decrease in malaria caused by *P. falciparum* in Oiapoque is related to the malaria elimination program launched by Suriname in 2009, which could reduce the burden of disease [28]. Suriname is a country that addresses the problem of mines through a program that seeks out miners to treat malaria: “looking for the gold, finding malaria” [29, 30]. This decrease was not seen in cases of *P. vivax*.

Timely diagnosis and effective treatment are part of strategies for eliminating *P. falciparum* in Brazil. Brazil has committed to eliminate malaria to advance with the Global Technical Strategy for Malaria 2016–2030. The first phase of the Brazilian plan consists of eliminating *P. falciparum* by 2019 [5]. The introduction of strains resistant to derivatives of artemisinin is a concern in Brazil. The municipality of Oiapoque receives Brazilian miners infected with *P. falciparum* from French Guiana or Suriname. Douine et al. (2016) [19] report that among miners in French Guiana, self-medication with derivatives of artemisinin was very high (53.7%), as was the non-adherence with the therapeutic schedule [31]. Poor adherence to treatment and together with the availability of low-quality medications are two main factors for the appearance of antimalarial resistance [32]. Although cases of antimalarial resistance have still not been identified in French Guiana, recently, a study found a resistant isolate through phenotypic methods with parasite survival rates above the threshold. This finding led to the speculation that the parasites may have reached a transient stage of resistance or tolerance to the medications [31].

Although complete information from Saint-Georges, a municipality in French Guiana, is not available from 2000 to 2012, cases of *P. vivax* have increased, and those of *P. falciparum* have decreased throughout the territory of French Guiana. The municipalities of Saint-Georges and Camopi are considered to have a low risk for malaria because of the seasonality of transmission [13]. The area along the Oiapoque and Camopi Rivers are of great epidemiological importance due to the presence of the miners along the entire border region. Data from Musset [13] show an incidence of 55.2 cases/1000 inhabitants in 2013 associated with Brazilian miners working illegally in French Guiana. On the Brazilian side of the border, in Ilha Bela, along with the route of the miners, there was an important number of cases in different moments of the study period.

Another change in the transmission dynamics observed over time was related to the profile of the people with the disease: in 2003, 74% of the ill were men; in 2015, although the majority of cases were among men, the percentage decreased to 55.9%, a statistically significant difference. Similarly, the percentage of young adults and those under 15 with malaria increased. The most affected age group was 10 to 14 years regardless of sex. These changes follow the national trend [33–35]. This data differs from what has been observed in areas of low transmission that have implemented malaria elimination programs. In Malaysia and Bhutan, for example, although the incidence of malaria has dramatically decreased, there has been an increase in the proportion of cases among young men and a decrease among those groups traditionally affected by the disease (i.e., women and children). It is assumed that the increase among these groups occurred because of the increased occupational risks faced by adult men when going to work in occupations in the forest or agricultural areas exposed to extradomiciliary mosquito bites [36].

## Conclusions

Our data point to the seasonality of the disease in Oiapoque. Both the graphs of cases per epidemiological period during the 13 years and the endemic curve clearly exhibit an increase in cases during the last trimester of each year. This information is essential for managers since measures to fight malaria should be intensified before the beginning of the period with the highest transmission. Malaria control in Amazonian municipalities has advanced day by day; however, many obstacles and challenges remain to overcome. The fragility of local health services continues to be an obstacle, and the dream of eliminating malaria in this region is still far from being reached [37]. Cooperation between health sectors and social actors in the municipality is essential. The support of the population and other actors, such as non-governmental organizations, universities, militaries, churches, and others, can strengthen surveillance and control and enable the elimination of malaria in the near future.

## Methods

### Study area

The municipality of Oiapoque is located in the extreme north of the state of Amapá (AP), Brazil, at a northern latitude of 03° 50′ 35″ and western longitude of 51° 50′ 06″, bordered by Saint-Georges and French Guiana (Fig. 6). Oiapoque has a population of 23,628 people [38] in an area of 22,625 km^2^. The city of Oiapoque originated as a military post in 1907 by the Brazilian government to protect the territory against the French. This post was later transferred to the location where Clevelândia do Norte is currently located. The municipality has a municipal capital, Oiapoque, and four districts: Clevelândia do Norte (an army post), Vila Velha (an agro-extractive settlement), Vila Brasil (serves as a support for mines infiltrated in French Guiana), and Taparabú (supports fishermen along the maritime coast). The municipality also includes environmental reserves: Cabo Orange Park and Tumucumaque Mountains Park.

In addition, it has indigenous lands, highlighting the Karipuna ethnic group in the villages within the Kumenê coverage area. There is only Manga coverage area, the Galibi Marworno, and Galibi in the Kumarumã and Palikur coverage areas, and the Palikur residing in one route connecting these areas to the state capital, Macapá: a road to the south of about 600 km with unpaved, narrow, and dangerous stretches, especially during the rainy season when many portions of the road are impassable because of quagmires. Both the distance to the capital and intense rains in the region limit traffic every year and create barriers to accessibility for the local population. The trip by bus from Macapá to Oiapoque lasts around 10 h during the dry season. Oiapoque is one of the poorest municipalities in the state of Amapá (10th poorest) with a human development index of 0.658 (considered low). The local economy is based on mineral extraction (mining), the gold trade, retail (Brazilian products sold to the Guyanese, especially meat and clothing), public services (with a major portion of the public payroll dedicated to the military), cassava cultivation, and vegetable extraction (especially of acai), in addition to the sale of horticultural products in the local market. Mining is illegal, but it boosts the local economy (trade, hotels, and bars). Subsistence fishing and hunting of several native species found in the rivers and forests of the region also exists. The sex trade frequently occurs in tandem with mining and weekend tourism activities. Cross-border interactions are harmful in this region, because of the need for Brazilians to hold visas to enter French Guiana versus the accessible entrance of Guianese into the Brazilian territory.

## Study design and data collection

### Seasonal analysis

Updated and scanned cartographic maps of the study area were obtained. Due to the absence of the Oiapoque municipal master plan, the polygons of the urban districts were constructed based on planning reports used by Google Earth Pro v. 7.3.0.3832 program. Thematic maps were elaborated with the Annual Parasite Incidence (API) grouped by risk level and year, according to the Ministry of Health guidelines for the epidemiological risk stratification of malaria.

The endemic curve was constructed utilizing the frequency of cases for every month within the studied period and done in two stages [39]. First, the averages and standard deviations of cases of every month during the 13 years of the study were calculated. The upper limit was calculated by adding standard deviations with the average, and the lower limit was calculated by subtracting the standard deviation from average cases. The months, which surpassed the upper limit of the average, were excluded from the initial analysis. During the second stage, the average and standard deviations were calculated again for each month after the exclusion of the “epidemic months.”

Additionally, a “control diagram” or “endemic curve” considered “normal” for this municipality was developed. Afterward, graphs with both the expected and observed number of cases were made. Months were considered an epidemic if they exceeded the expected upper limit (Additional file 2).

#### Statistical analysis

All the data was stored and analyzed using Epi Info v.6.0 (Centers for Disease Control and Prevention, Atlanta, GA, USA). Descriptive analysis was undertaken, and the chi-squared test (*χ*^2^) was utilized to analyze differences in proportions of sex, age, season, and parasite species. The *t* test was used to compare averages. A *p* value < 0.05 was considered statistically significant.

## Additional files

Additional file 1: Geographic location of the Polos Base within the municipality of Oiapoque-AP Brazil. (PNG 244 kb)

Additional file 2: Epidemiological database of the municipality of Oiapoque- Ap Brazil. (XLSX 47 kb)
