# Supplementary figures and images for: Complex malaria epidemiology in an international border area between Brazil and French Guiana: challenges for elimination
Source: Trop Med Health. 2019 Apr 11;47:24. doi: 10.1186/s41182-019-0150-0 (PMC6458633; doi:10.1186/s41182-019-0150-0)

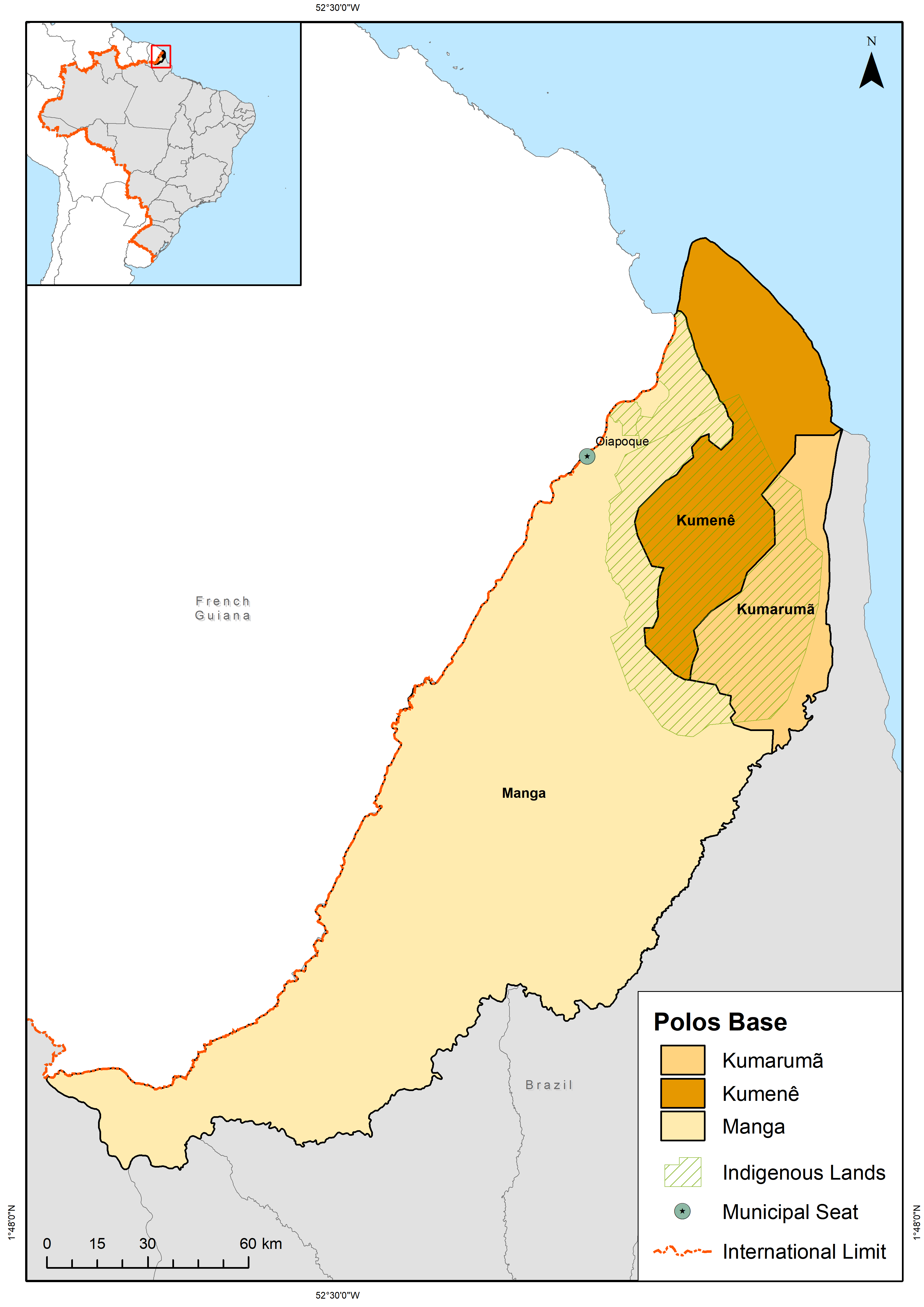

Supplement: Supplementary file 1 — Geographic location of the Polos Base within the municipality of Oiapoque-AP Brazil. (PNG 244 kb) [file 41182_2019_150_MOESM1_ESM.png]
